# Incidental Olfactory Groove Meningioma: A Case Report

**DOI:** 10.7759/cureus.85470

**Published:** 2025-06-06

**Authors:** Hani Aljohani, Abdulrahman H Alashkar, Mohammad A Aljawash, Salah A Kassab

**Affiliations:** 1 College of Medicine, Qassim University, Buraidah, SAU; 2 Surgery, Dr. Sulaiman Al-Habib Medical Group, Buraidah, SAU

**Keywords:** anxiety, headache, meningioma, olfactory groove, skull base

## Abstract

Olfactory groove meningiomas (OGMs) are midline meningiomas of the anterior cranial fossa. They often grow to large sizes before producing symptoms. This, together with the increasing use of neuroimaging, has made diagnosing OGMs incidentally a more likely scenario. Due to the position of OGMs in relation to the frontal lobe, these tumors are likely to cause neuropsychiatric symptoms. In this report, we present a 41-year-old female who was diagnosed with incidental OGM and focus on the patient’s perspective; she reported anxiety and frontal headaches only after being diagnosed with the tumor.

## Introduction

In adults, meningiomas are the most common primary tumors of the central nervous system (CNS). They originate from arachnoid cap cells, most commonly at the brain convexities and the parasagittal area. Olfactory groove meningiomas (OGMs) are midline meningiomas of the anterior cranial fossa. They arise from the meningeal coverings of the ethmoid lamina cribrosa and represent 8-13% of all intracranial meningiomas. Unlike other midline meningiomas of the anterior cranial fossa, OGMs often grow to large sizes before manifesting clinically [1,2]. This, together with the increasing use of neuroimaging, has made diagnosing OGMs incidentally a more likely scenario. The diagnosis of a tumor, in this case a meningioma, could understandably cause anxiety to patients, which could prompt them to request surgical excision instead of surveillance even if the meningioma is asymptomatic and benign-looking. This can cause a dilemma for the treating surgeon as to whether or not to offer surgical treatment [3]. The situation with an OGM can be more complex, as it’s known to cause personality and mental changes due to its position in relation to the frontal lobe [4]. Here, we present a case of incidentally diagnosed OGM in which the patient requested surgical resection despite the tumor being benign-looking.

## Case presentation

A 41-year-old medically free woman presented to the neurosurgery clinic due to an intracranial frontal mass lesion diagnosed incidentally about five months earlier when she had a computed tomography (CT) scan at another hospital. Prior to being scanned, she had no complaints that could be attributed to the mass lesion. However, after she had been informed about the CT findings, she insisted that she “started to feel something behind her eyes” and that it was “causing her to have headaches and anxiety.”

**Figure 1:**
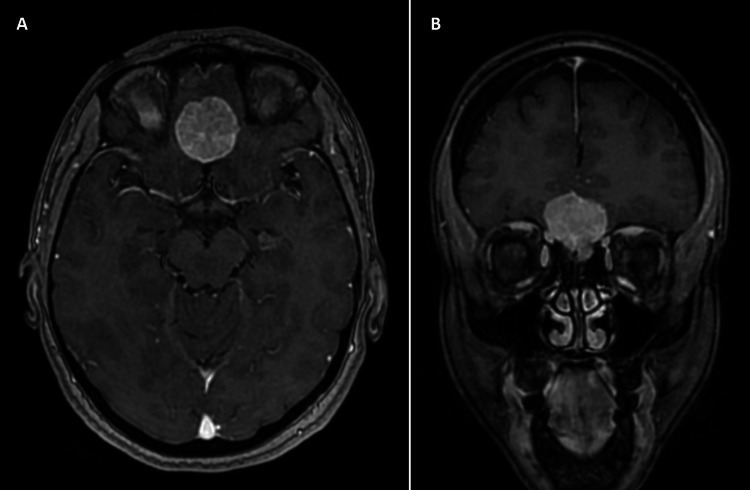
Preoperative MRI Preoperative gadolinium-enhanced T1-weighted images in axial (A) and coronal (B) views showing a well-demarcated and homogenously enhancing mass lesion situated at the midline of the anterior fossa of the skull base.

On examination, the patient was conscious, oriented, and vitally stable. She did not have any focal neurological deficits, and her cranial nerves were clinically intact. Gadolinium-enhanced MRI of the brain revealed an extra-axial and well-demarcated mass lesion at the midline of the anterior fossa of the skull base with a maximum diameter of just under 3 cm (2.8 x 2.5 x 2.4 cm). Accordingly, a provisional diagnosis of OGM was made (Figure 1).

**Figure 2:**
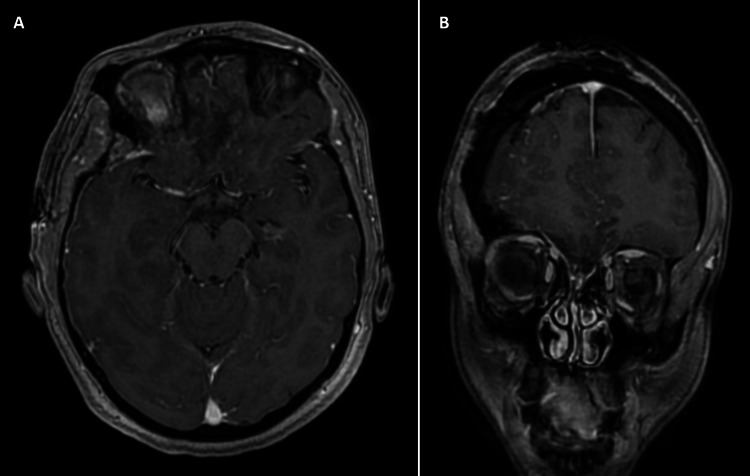
Postoperative MRI Postoperative gadolinium-enhanced T1-weighted images in axial (A) and coronal (B) views showing complete resection of the mass lesion.

The patient was counseled about treatment options and risks, and she requested surgical excision of the mass lesion. She underwent microscopic excision of the mass lesion using right pterional craniotomy. Postoperatively, MRI confirmed total resection of the lesion (Figure 2), which was shown upon histopathological assessment to be a benign (WHO grade 1) meningioma.

Also, postoperative assessment revealed no neurologic deficits, and she reported having a better sense of well-being. She was discharged in a stable condition on the seventh postoperative day. The patient was reviewed one month following discharge, during which she was stable and satisfied with the outcome of the surgery. She was advised to repeat the MRI after one year.

## Discussion

Meningiomas are the most common primary tumors of the CNS in adults, and OGMs represent 8% to 13% of all intracranial meningiomas [2]. Expectedly, meningiomas are also the most common primary benign tumors of the brain to be diagnosed incidentally. In a population-based study that assessed the prevalence of incidental brain findings on MRI, primary benign tumors were diagnosed in 1.6%, and benign meningiomas in 0.9%, with a prevalence of 1.1% and 0.7% in females and males, respectively [5].

**Figure 3:**
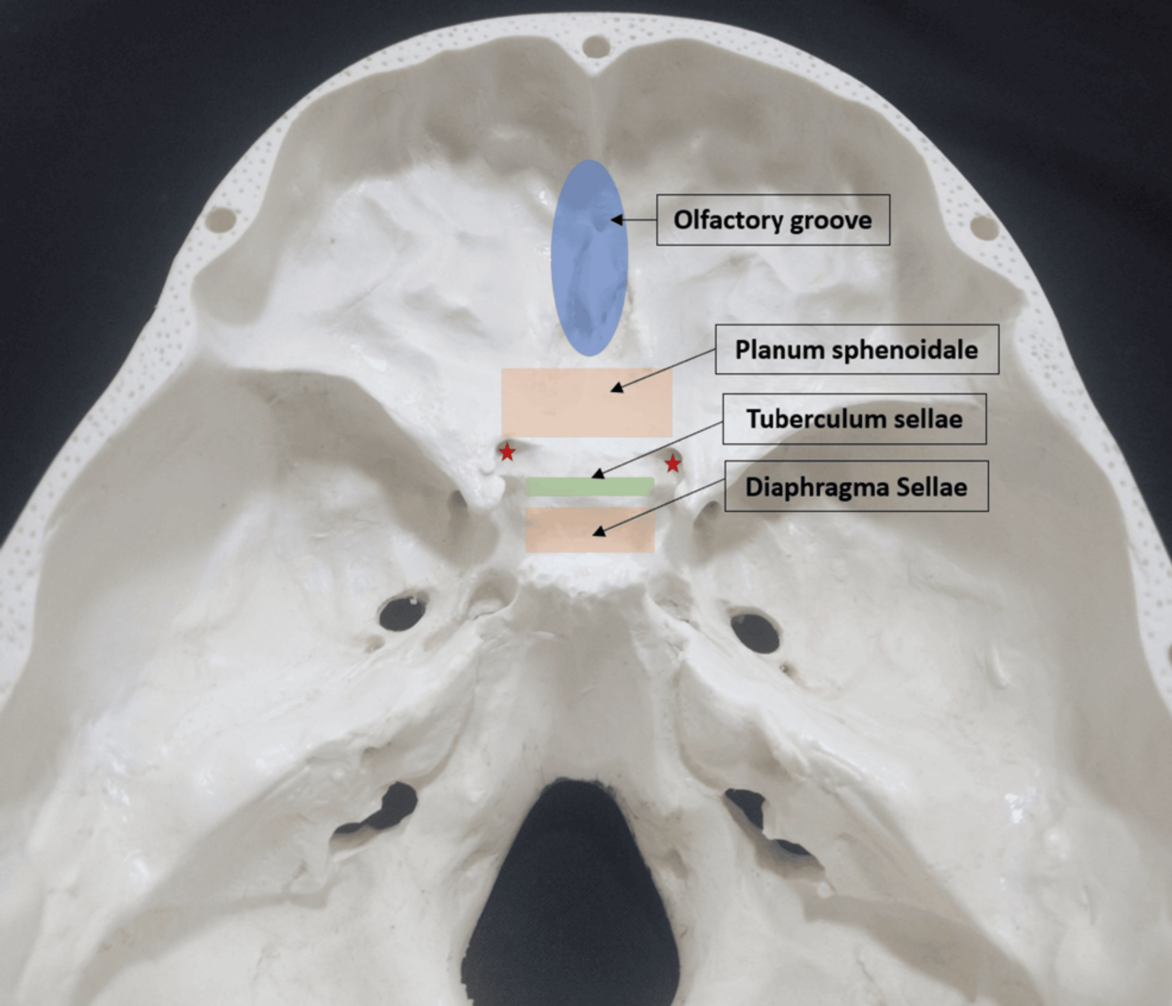
Anterior skull base A model of the skull base showing the approximate locations of the midline anterior skull base meningiomas and demonstrating their relative positions to the optic pathways (red stars mark the openings of the optic canals). Reference: [6] Image Credits: Dr. Abdulrahman H. Alashkar

OGMs, together with suprasellar meningiomas (planum sphenoidale, tuberculum sellae, and diaphragma sellae meningiomas), represent the midline meningiomas of the anterior skull base. Of them, OGMs are positioned most anteriorly, originating from the meningeal covering of the cribriform plate (Figure 3). Compared to suprasellar meningiomas, OGMs are the farthest from the optic pathways and the least likely to cause neurological deficits at smaller sizes [6], which allows OGMs to grow to formidable sizes before manifesting clinically. This, together with the increasing use of neuroimaging, has made discovering OGMs incidentally a more likely scenario.

Although histological assessment is the gold-standard diagnostic tool, an OGM has a very characteristic appearance on MRI. It appears as an extra-axial and well-demarcated mass lesion at the midline of the anterior fossa of the skull base. It is iso- to slightly hypointense to gray matter on T1-weighted images, iso- to hyperintense on T2-weighted images, and shows homogeneous enhancement with gadolinium [7].

When diagnosed incidentally, the patient and the surgeon are faced with several questions. Such questions include: “what is the ideal initial management?”, “what is the expected growth rate?”, "what are the surgical risks" and “is surgery riskier if performed at a later stage?” While these questions seem intuitive, their answers might not be straightforward. This is due to the lack of sufficient evidence and the answers being largely dependent on the surgeon’s personal experience [3].

Moreover, simply knowing about the tumor can cause significant anxiety and distress to the patient, which could further complicate decision-making. Incidental imaging findings caused moderate to severe levels of psychological distress to 28.6% of patients in a survey that included 471 patients who had undergone whole-body MRI [8]. Indeed, the patient in this case indicated symptom development only after she had been informed about the mass lesion. She insisted that she started to feel the tumor in her head and that it was causing her to have headaches.

Although the management of incidental meningiomas is still debatable, active surveillance is generally the first-line treatment [9]. Nevertheless, OGMs are known to cause neuropsychiatric changes due to their location in relation to the frontal lobe [10,11], and abnormal neuropsychologic testing should prompt surgical treatment of an otherwise incidental OGM [12]. So, surgery was an option for our patient, whether her complaints were real organic symptoms or psychogenic and secondary to the tumor’s effect on the frontal lobe.

Several studies have reported the absolute (AGR) and relative growth rate (RGR) of incidental meningiomas. Nakamura et al. reported the natural history of incidental meningiomas in 41 patients who were managed conservatively. They reported a mean AGR and a mean RGR of 0.796 cm^3^/year (range: 0.03-2.62 cm^3^/year), and 14.6%/year (range: 0.48-72.8%/year), respectively [13]. Of note, pregnancy has been shown to accelerate meningioma growth due to hormonal factors, an observation that needs to be taken into account when managing meningiomas in females of childbearing age [14].

When surgical treatment is chosen, the risks include anosmia, bleeding, infections, and cerebrospinal fluid leakage, and the incidence of each largely depends on the tumor characteristics and surgeon's experience [15]. On the other hand, when active surveillance is opted for, the likelihood that intervention will be needed at some point depends on multiple factors, such as the tumor location, the development of symptoms, and the patient’s fitness. Nevertheless, it should be kept in mind that for OGMs, mortality and morbidity of surgical intervention increase significantly when the tumor is greater than 3 cm in diameter [16].

So, for our patient, considering her complaints of anxiety and headache, the fact that she is still in her child-bearing age, the tumor size being just under 3 cm, and her personal preference for surgical excision, surgery was arguably the best initial treatment for her.

Lastly, although no formal psychological assessment was done for this case, it is advisable for such cases.

## Conclusions

Among the midline anterior skull base meningiomas, OGMs are the most likely to grow to formidable sizes before manifesting clinically. Because of this, together with the increasing use of neuroimaging, more OGMs are diagnosed incidentally. OGMs can lead to the development of neuropsychiatric symptoms, which is an indication of intervention. Although the management of incidental meningiomas is still debatable, active surveillance is generally the first-line treatment. Nevertheless, several factors need to be taken into consideration when deciding the best initial management of an incidental OGM, including the patient’s preference, age, and the tumor’s size.

## References

[1] Mirimanoff RO, Dosoretz DE, Linggood RM, Ojemann RG, Martuza RL (1985). Meningioma: analysis of recurrence and progression following neurosurgical resection. J Neurosurg.

[2] Nakamura M, Struck M, Roser F, Vorkapic P, Samii M (2007). Olfactory groove meningiomas: clinical outcome and recurrence rates after tumor removal through the frontolateral and bifrontal approach. Neurosurgery.

[3] Islim AI, Millward CP, Mills SJ (2023). The management of incidental meningioma: an unresolved clinical conundrum. Neurooncol Adv.

[4] Pallini R, Fernandez E, Lauretti L (2015). Olfactory groove meningioma: report of 99 cases surgically treated at the Catholic University School of Medicine, Rome. World Neurosurg.

[5] Vernooij MW, Ikram MA, Tanghe HL (2007). Incidental findings on brain MRI in the general population. N Engl J Med.

[6] Ajlan A, Noor Elahi B, Almeshari S (2024). Suprasellar meningioma classification: endoscopic transnasal perspective. J Neurol Surg B Skull Base.

[7] Adappa ND, Lee JY, Chiu AG, Palmer JN (2011). Olfactory groove meningioma. Otolaryngol Clin North Am.

[8] Schmidt CO, Hegenscheid K, Erdmann P (2013). Psychosocial consequences and severity of disclosed incidental findings from whole-body MRI in a general population study. Eur Radiol.

[9] Goldbrunner R, Stavrinou P, Jenkinson MD (2021). EANO guideline on the diagnosis and management of meningiomas. Neuro Oncol.

[10] Ravikanth R, Pinto DS, Mathew S (2018). Olfactory groove meningioma masquerading as psychiatric disturbances. Indian J Psychiatry.

[11] Leo RJ, DuBois RL (2016). A case of olfactory groove meningioma misdiagnosed as schizophrenia. J Clin Psychiatry.

[12] DeMonte F, Raza SM (2020). Olfactory groove and planum meningiomas. Handb Clin Neurol.

[13] Nakamura M, Roser F, Michel J, Jacobs C, Samii M (2003). The natural history of incidental meningiomas. Neurosurgery.

[14] Giraldi L, Lauridsen EK, Maier AD (2021). Pathologic characteristics of pregnancy-related meningiomas. Cancers (Basel).

[15] Mukherjee S, Thakur B, Corns R, Connor S, Bhangoo R, Ashkan K, Gullan R (2015). Resection of olfactory groove meningioma - a review of complications and prognostic factors. Br J Neurosurg.

[16] Greenberg MS (2019). Handbook of Neurosurgery. Handbook of Neurosurgery. Thieme Publishers, New York, NY.

